# Clinical Nasal Deviation Following Midface Advancement in Patients With Syndromic Craniosynostosis

**DOI:** 10.1097/SCS.0000000000011186

**Published:** 2025-03-27

**Authors:** Iris E. Cuperus, Parinaz Rostamzad, Simone E. Bernard, Sarah L. Versnel, Laura L. Veder, Irene M.J. Mathijssen

**Affiliations:** * Department of Plastic and Reconstructive Surgery and Hand Surgery, Sophia Children’s Hospital - Erasmus Medical Center, Rotterdam, the Netherlands; † Department of Otorhinolaryngology, Sophia Children’s Hospital - Erasmus Medical Center, Rotterdam, The Netherlands

**Keywords:** Apert syndrome, craniosynostosis, Crouzon syndrome, Le Fort III, monobloc, midface surgery, nasal deviation, syndromic craniosynostosis

## Abstract

**Introduction:**

Nasal deviations have been observed in patients following midface surgery. Therefore, the purpose of this study was to evaluate the development of clinically visible nasal deviations following midface surgery and to assess the natural course of the nose over time.

**Methods:**

This retrospective study included all Apert and Crouzon patients who underwent midface surgery (Le Fort III (LF3), monobloc (MB), or facial bipartition (FB)). Clinical nasal deviation was assessed on preoperative, short-term postoperative (≤ 1 year), and long-term postoperative (> 1 year) 2D facial photographs by consensus of pediatric ENT surgeons and one craniofacial surgeon. Additionally, pre- and postoperative CT scans were reviewed when available.

**Results:**

Sixty-eight procedures were included (27 Apert, 41 Crouzon); 34 LF3, 31 MB, and 3 FB. The median age at surgery was 10.2 years. Twenty-five (37%) patients were found to have clinically worsening nasal asymmetry, of whom 4 (16%) had pre-existing deviation preoperatively. Seventeen patients with worsening deviation had long-term postoperative facial photographs available (median time 5.8 years), and in 9 (53%), the postoperative clinical nasal deviation appeared to improve spontaneously. We were unable to identify predictive factors for postoperative nasal deviation.

**Conclusion:**

In 16% of the patients, a clinically deviated nose was observed preoperatively. Nasal deviation worsened in 37% of the patients after midface surgery, but also spontaneously improved in 53% of these patients over the long-term. Since predicting the occurrence of nasal deviation and self-correction is difficult, the possibility of developing nasal deviation should be discussed with the patient and their parents.

Patients with Apert and Crouzon syndromes are known to suffer from (severe) midface hypoplasia. This can lead to both functional problems (e.g., obstructive sleep apnea, exorbitism, malocclusion) and aesthetic complaints and may require surgical correction.^1,2^ The different types of midface advancement used to treat midface hypoplasia are Le Fort III (LF3), a variant of this procedure (such as Le Fort II with simultaneous zygomatic repositioning), a monobloc (MB), or facial bipartition (FB), usually combined with distraction. The LF3 is performed to enhance the midface, correct malocclusion, correct the position of the zygomatic bones and nose, and improve facial aesthetics.^3^ MB is used to additionally treat exorbitism and augment intracranial volume by advancement of the forehead and supraorbital rims.^4,5^ FB is advised for correcting hypertelorism and a V-shaped alveolar arch.^6^

Nasal deviation following midface surgery has been clinically observed in patients treated at our craniofacial center. In addition, in some of our patients, the nasal deviation appears to improve over time without the need for corrective surgery. The purpose of this study was to determine the extent to which patients develop clinically visible nasal deviation following midface advancement, to evaluate the occurrence of self-correction, and to identify predictors of postoperative nasal deviation.

## METHODS

This retrospective study was approved by the medical ethics committee of the Erasmus MC (MEC-2016-312). All Apert and Crouzon patients treated at the Sophia Children’s Hospital in Rotterdam between 1998 and 2023 with a LF3, MB, or FB were eligible for inclusion. Patients had to have standardized preoperative and postoperative frontal view 2D facial photographs within one year of surgery to be included in this study.

### Surgical Procedure

The type of midface advancement was mainly dependent on individual facial anomalies. Given the severity of midface hypoplasia in both syndromes, there is usually an indication for distraction. During the procedure, the midline osteotomy of the ethmoid and the septum/vomer is performed using an osteotome all the way down to the vomer. In our center, rhinoseptoplasty is preferably postponed until skeletal maturity in order not to interfere with facial growth and to limit recurrent deviations.

### Outcome Variables and Data Collection

Clinical nasal deviation was assessed on preoperative, short-term postoperative (≤ 1 year after surgery) (T1), and long-term postoperative (> 1 year after surgery) (T2) 2D facial photographs. In addition to the frontal view 2D photographs, bottom (worm’s eye) and/or top (bird’s eye) view photographs were collected if available. On the preoperative photographs, the nasal deviation was scored as either absent or present. On the short- and long-term facial photographs, nasal deviation was scored as unchanged, worsened, or improved. The short-term photograph was compared to the preoperative photograph, and the long-term photograph was compared to the short-term photograph. All photographs were reviewed by two pediatric ENT surgeons and one craniofacial plastic surgeon and were scored by consensus among the three specialists. Patients were excluded if consensus could not be reached regarding the short-term postoperative position. Postoperative facial photographs that were taken after a rhinoplasty or other orthognathic/midface surgery that may have affected nasal position were also excluded. Any patients who sustained facial trauma postoperatively were excluded. Demographic (gender, diagnosis, age) and surgical data (type of surgery, type of distraction, intraoperative complications, postoperative rhinoplasty) were collected from patients records. In addition, preoperative, the first postoperative (T3), and long-term (T4) postoperative computed tomography (CT) scans were reviewed when available. The scans were evaluated for (changes in) anterior and posterior nasal septum deviations.

### Statistical Analysis

Data were imported into R statistical software (version 4.1.2, R Foundation for Statistical Computing) for analysis. Histograms and QQ-plots were used to assess the distribution of continuous variables. Normally distributed continuous data are presented as means with standard deviation and skewed data as medians with interquartile ranges (IQR). Categorical data were presented as counts and proportions. Descriptive statistics were used only.

## RESULTS

### Study Population

After excluding three patients in whom consensus was not reached and four patients in whom postoperative facial photographs were taken after other orthognathic/midface surgery, 68 surgeries in 66 patients were included (Supplemental Table 1, Supplemental Digital Content 1, http://links.lww.com/SCS/H449). No patients sustained facial trauma postoperatively. Thirty-four (50%) patients had received a LF3, 31 (46%) MB, and 3 (4%) FB. The median age at surgery was 10.2 years old (IQR 6.9 – 17.7).

An external frame only was used in 20 (59%) patients with a LF3, and internal distraction was used in 31 patients (90%) with MB. Two out of three FB patients received both internal distraction and an external frame. In 11 (16%) patients a pre-existing nasal deviation was clinically observed.

### Clinical Nasal Deviation ≤ 1 Year Postoperatively

**FIGURE 1:**
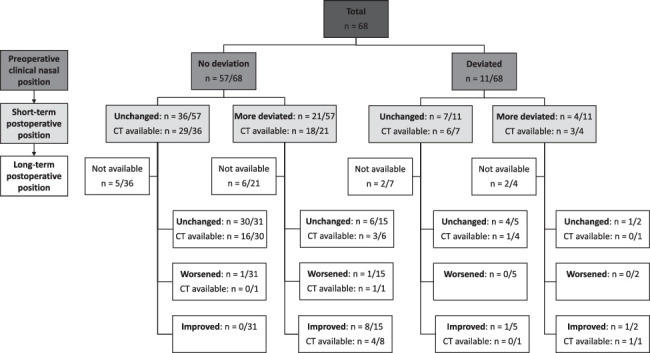
Flowchart showing short-term and long-term postoperative clinical nasal deviation per preoperative clinical position.

Figure 1 demonstrates the short- and long-term postoperative clinical nasal deviation per preoperative clinical position. A total of 25 patients (37%) showed an increased deviation postoperatively; 21 developed new deviation, while 4 experienced worsening of a pre-existing deviation (Fig. 1). Based on the photographs of these 25 patients, 18 had a clinically mild deviation and 7 had a severe deviation. Two patients (ages 2.9 and 9.5 years) had such severe deviations that they required septoplasty within a few months of midface surgery. No children showed improvement in nasal deviation in the short-term postoperatively. There was no evidence that diagnosis, preoperative clinical septum deviation as seen on the head CT scan, previous midface surgery, type of midface surgery, or age at surgery were associated with postoperative nasal deviation, as there was no difference in these variables between patients with unchanged or worsened nasal asymmetry (Supplemental Table 1, Supplemental Digital Content 1, http://links.lww.com/SCS/H449).

### Clinical Nasal Deviation > 1 Year Postoperatively

Details on the long-term postoperative nasal deviation per preoperative and short-term postoperative deviation are given in Fig. 1. The median time between surgery and the long-term photography was 5.6 years (IQR 3.2 – 8.8) (Supplemental Table 2, Supplemental Digital Content 1, http://links.lww.com/SCS/H449). Fifty-three patients had long-term postoperative facial photographs available. The clinical nasal position remained unchanged in 41 (77%) patients (30 had no deviation short-term postoperatively and 11 had a persistent deviation). Two patients showed a worsening of the nasal position (1 developed a new deviation on the long-term photograph and 1 had a further worsening of the deviation compared to the short-term photograph). Ten patients showed improvement in nasal deviation compared to the short-term postoperative photographs (9 patients with a more deviated nose on the short-term photograph and 1 patient with an unchanged position of a preoperative pre-existing deviation on the short-term photograph). Details of the patient characteristics of 9 patients who showed an improvement in nasal deviation in the long-term photograph compared to the worsening observed in the short-term photograph are provided in Supplemental Table 3, Supplemental Digital Content 1, http://links.lww.com/SCS/H449. Self-correction of short-term postoperative nasal deviation was also observed in 4 patients with severe deviation.

### Assessment of the Nasal Septum Deviation on CT Scan

Fifty-six patients had preoperative and short-term postoperative CT scans of the head (Fig. 1); 47 (84%) patients without preoperative nasal deviation and 9 (16%) with preoperative deviation based on the facial photographs. The median time between surgery and first postoperative CT scan was 4.0 months (IQR 3.0 – 7.5) (Supplemental Table 4, Supplemental Digital Content 1, http://links.lww.com/SCS/H449). Twenty-six patients had long-term postoperative CT scans available; 17 (65%) patients with unchanged nasal deviation on the short-term postoperative photograph and 9 (35%) with worsened deviation on the short-term postoperative photograph. The median time between surgery and the last postoperative CT scan was 4.5 years (3.0 – 8.8).

**FIGURE 2:**
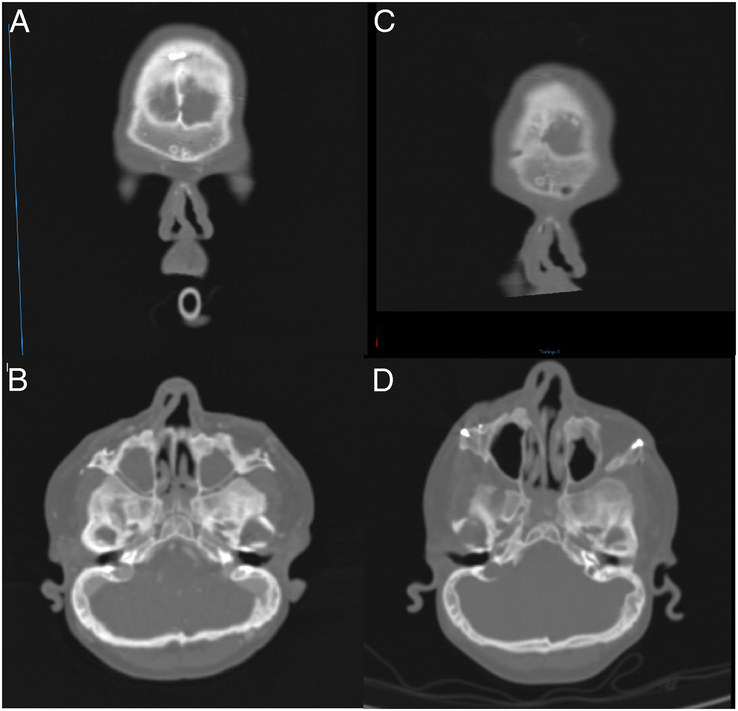
A-D Patient underwent MB with internal distractors at the age of 12. Clinically, she had no deviation of the nose preoperatively, and the nasal position was unchanged in the short-term postoperative photograph. However, both the preoperative and short-term postoperative CT scans of the head showed a pre-existing deviation and a worsening of the deviation, respectively: A. preoperative frontal view showing a C-shaped septal deviation anterior to the ANS, B. preoperative axial view showing a straight external nose and bony septum, but deviated cartilaginous septum, C. 9 moths postoperative frontal view showing a worsened C-shaped septal deviation anterior to the ANS, D. 9 moths postoperative axial view showing a similar image to the preoperative situation.

A clinical deviation on the facial photograph did not always correspond to a deviation on the CT scan, and vice versa. In 26 (55%) patients without preoperative clinical nasal deviation on the facial photograph, an anterior nasal septum deviation was observed on the preoperative CT scan: a cephalocaudal C-shaped deviation in 12 (46%) (Fig. 2 A-B), a displacement of the caudal septum in 11 (42%), and a septal spur in 3 (12%). Of the 9 patients with preoperative clinical nasal deviation, 6 had a cephalocaudal C-shaped deviation and 3 had a displacement of the caudal septum.

**FIGURE 3:**
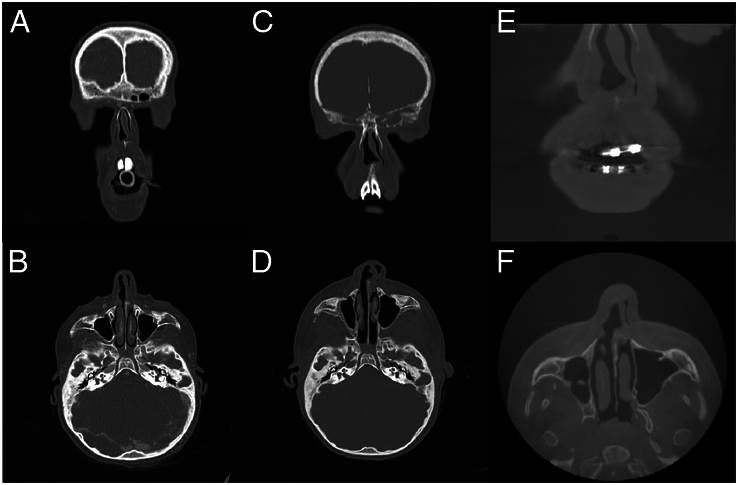
A-F Example of a patient with worsened nasal asymmetry postoperatively. Patient underwent MB with internal distractors at 10 years old. Clinically, preoperatively a clinically deviated nose was observed, on the short-term postoperative facial photograph a worsened deviation was observed, and on the long-term facial photograph an improvement of the nasal deviation was observed. The clinical findings were align with the findings on the CT scans: A. preoperative frontal view showing a small caudal septal dislocation at the level of the ANS, B. preoperative axial view showing the preoperative position, C. 4 month postoperative frontal view showing an increased caudal septal dislocation, D. 4 months postoperative axial view showing a dislocation of the cartilaginous septum and flattening of the external nose, E. 4 years postoperative frontal view showing a slightly reduced caudal septal dislocation without surgery, F. 4 years postoperative axial view still showing dislocation of the cartilaginous septum but improved position of the external nose.

Of the 21 patients with a worsened nasal deviation on the short-term facial photograph (18 with postoperative new onset deviation and 3 with worsened deviation of the pre-existing deviated nose), 10 (48%) had a distinct different position of the anterior septum on the postoperative CT scan; 8 patients had an increased cephalocaudal C-shaped septum deviation starting at the level of the anterior nasal spine (ANS), and 2 patients had an increased displacement of the caudal septum at the level of the ANS (Fig. 3 A-D). In the other 11 (52%) patients with worsened nasal deviation on the short-term facial photograph, no changes in the anterior septum were found on the postoperative CT scan.

Of the 35 patients with unchanged nasal position on the short-term facial photograph, in 6 (17%) changes in the position of the anterior septum were seen on the postoperative CT scan; 5 patients showed an increased C-shaped septum deviation (Fig. 2 C-D), and 1 had a caudal dislocation. In the remaining 29 patients (83%), no change in the anterior septum was seen on the short-term postoperative CT. Of the 29 patients without postoperative changes, 13 (45%) had a straight septum, 6 (21%) had a caudal dislocation, and 10 (34%) had a C-shaped deviation preoperatively. Furthermore, of the 56 patients with available short-term postoperative CT scans, an increased deviation of the posterior septum was noted in 4 (7%) patients.

Self-correction of nasal deviation was also seen on postoperative long-term CT scans (Fig. 3 E-D). Five patients with clinically improved nasal deviation on the long-term facial photograph had long-term CT scans available (Fig. 1), and in 3 of these patients this improvement was also evident on the long-term postoperative CT-scan.

## DISCUSSION

This study evaluated clinical nasal deviation following midface advancement in a population of patients with Apert and Crouzon syndrome. In addition, it aimed to evaluate the occurrence of self-correction over time and to identify predictors of postoperative nasal deviation. The results showed that 16% of the patients had a clinically deviated nose prior to surgery. Within one year of surgery, nasal deviation was observed in 37% of the patients, the majority without a preoperative deviation.

Furthermore, during long-term follow-up with a median of 5.6 years, 53% of the patients who showed an increase in deviation short-term postoperatively, showed spontaneous improvement without additional surgery. This finding suggests a potential for self-correction. Within our cohort, two patients underwent septoplasty within one year of surgery to correct the severe deformity that was caused by midface advancement. Moreover, in this study two types of nasal septum deviation were frequently identified on postoperative CT scans; an increased cephalocaudal C-shaped deviation of the anterior septum and a luxation of the caudal septum at the level of the ANS. Changes of the posterior part of the septum were uncommon.

This study demonstrated that there is no evidence to suggest a clear association between postoperative nasal septum deviation and factors such as preoperative clinical septum deviation, type of midface surgery, diagnosis, age at surgery, or previous midface surgery.

Children with Apert and Crouzon syndromes often present with nasal/nasopharyngeal obstruction due to small nasal cavities and nasal septum deviation.^7,8^ However, the literature on the effect of midface surgery on nasal septum deviation in these patients is limited. One case study reported nasal septum deviation in one of their patients following a LF3 procedure, necessitating additional corrective rhinoplasty.^9^ Our study indicates the likelihood of developing a nasal deviation following midface advancement and its natural course over time, which is relevant information for the patients and parents. It remains unpredictable which patients will experience postoperative worsening of nasal deviation and which will experience spontaneous improvement, as both have been seen in patients with either Apert or Crouzon syndromes, after all midface advancement procedures, in patients with and without pre-existing nasal deviations, and in patients of different ages. This emphasizes the importance of proper counseling of the patient and their parents.

We can only speculate about the mechanism behind the occurrence of nasal deviation and the self-correction, and therefore we cannot advise on how to avoid nasal deviation. In clinical practice, we notice that the nasal deviation occurs halfway through the distraction process. This may indicate that the increased free space for the released posterior edge of the septum allows warping of the cartilage.

This study had some limitations. First, we used only a subjective method to quantify nasal deviation because we could not find an easily reproducible, more objective method. We considered numerous previously used methods to objectively quantify nasal septum changes on CT scans, but many lacked clear clinical interpretation or were difficult to reproduce and lead to inconsistent outcomes, especially in postoperative scans and in this patient population with pre-existing abnormal anatomy.^10–15^ We then tried a 3D analysis, creating a 3D soft-tissue MESH from the preoperative scan and aligning it with the postoperative scan using Mimics software (version 25.0, Materialise, Belgium). We used R3DS WRAP software (version 3.4, Faceform LLC, Armenia) to manually place three landmarks (nasion, pronasale, subnasale) to analyze clinical changes in the nose. In practice, we found that the margin of error and reproducibility of this analysis were as large as the measured deviation, making it an unreliable method. This, along with a relatively small study population with limited statistical power, ultimately led us to our subjective method of quantifying nasal deviation. The fact that there is no single most commonly used method for this seemingly simple task already indicates its difficulty, and future research should aim to use novel 3D and AI technologies to more objectively measure preoperative and postoperative nasal septum changes. Additionally, not all patients had “worms eye” or “bird’s eye” photographs available, which potentially limited looking at symmetry. Furthermore, the presence of functional symptoms was not routinely assessed during follow-up at our center, and therefore the (long-term) functional impact of the postoperative anterior septum deviation could not be determined. The same is true for the clinical impact of the increased posterior septum deviation found in four patients. Prospective studies should focus on assessing functional symptoms and correlate them with the observed deviations.

## CONCLUSION

In conclusion, in 16% of the patients, a clinically deviated nose prior to surgery was observed. In addition, clinical nasal deviation worsened in 37% of the patients shortly after midface advancement, but also spontaneously improved in 53% of these patients over the long-term. Since it is currently impossible to predict who will develop nasal deviation and who will experience self-correction, we recommend that each patient and parents are made aware of the potential risk of worsened nasal septum deviation.

## Supplementary Material

SUPPLEMENTARY MATERIAL
