# Supplementary material for: Clinical Nasal Deviation Following Midface Advancement in Patients With Syndromic Craniosynostosis
Source: J Craniofac Surg. 2025 Mar 27;36(5):1552–5. doi: 10.1097/SCS.0000000000011186 (PMC12187153; doi:10.1097/SCS.0000000000011186)
Supplement: SUPPLEMENTARY MATERIAL [file scs-36-1552-s001.docx]

**Supplemental Table 1. Patient characteristics.**

|  |  | **Short-term postop clinical nasal deviation** | |
| --- | --- | --- | --- |
|  | **Total** | **Unchanged** | **More deviated** |
| **No. patients** | 68 | 43 | 25 |
| **Males : females** | 34 : 34 (50 : 50) | 28 : 15 (65 : 35) | 6 : 19 (24 : 76) |
| **Diagnosis** |  |  |  |
| Apert | 27 (40) | 17 (40) | 10 (40) |
| Crouzon | 41 (60) | 26 (60) | 15 (60) |
| **Preop clinical nasal position** |  |  |  |
| No deviation | 57 (84) | 36 (84) | 21 (84) |
| Deviated | 11 (16) | 7 (16) | 4 (16) |
| **Previous midface surgery** | 9 (13) | 6 (14) | 3 (12) |
| **Median age at surgery (IQR), yrs** | 10.2 (6.9 – 17.7) | 9.7 (6.7 – 16.1) | 10.8 (6.9 – 17.7) |
| **Type of surgery** |  |  |  |
| LF3 | 34 (50) | 23 (53) | 11 (44) |
| MB | 31 (46) | 19 (44) | 12 (48) |
| FB | 3 (4) | 1 (2) | 2 (8) |
| **Type of distraction** |  |  |  |
| No distraction | 7 (10) | 3 (7) | 4 (16) |
| Internal | 31 (46) | 18 (42) | 13 (52) |
| External | 21 (31) | 15 (35) | 6 (24) |
| Internal + external | 9 (13) | 7 (16) | 2 (8) |
| **Median T1 (IQR), mos** | 3.0 (1.0 – 5.0) | 3.5 (2.0 – 6.0) | 3.0 (1.0 – 4.0) |

Values represent number of patients (percentages).

Preop = preoperative, postop= postoperative, LF3 = Le Fort III, MB = monobloc, FB = facial bipartition, IQR = interquartile range, T1 = short-term postoperative (≤ 1 year after surgery) 2D facial photograph.

**Supplemental Table 2. Long-term nasal deviation compared to short-term postoperative photographs.**

|  |  | **Short-term postop clinical nasal deviation** | |
| --- | --- | --- | --- |
|  | **Total** | **Unchanged** | **More deviated** |
| **No. of patients** | 53 | 36 | 17 |
| **Median T2 (IQR), yrs** | 5.6 (3.2 – 8.8) | 5.9 (3.2 – 8.8) | 5.8 (3.3 – 8.4) |
| **Long-term postop clinical nasal deviation** |  |  |  |
| Unchanged | 41 (77) | 34 (94) | 7 (41) |
| Worsened | 2 (4) | 1 (3) | 1 (6) |
| Improved | 10 (19) | 1 (3) | 9 (53) |

Values represent number of patients (percentages).

Postop = postoperative, IQR = interquartile range, T2 = long-term postoperative (>1 year after surgery) 2D facial photographs

**Supplemental Table 3. Patient characteristics of patients who had a more deviated nose on the short-term postoperative facial photograph, but whose deviation had improved on the long-term facial photographs.**

| # | **Diagnosis** | **Age of surgery** | **Type of surgery** | **Type of distraction** | **Clinical nasal deviation preop** |
| --- | --- | --- | --- | --- | --- |
| 1 | Apert | 11.9 | LF3 | External | No |
| 2 | Apert | 4.3 | MB | Internal | No |
| 3 | Apert | 13.8 | LF3 | External | No |
| 4 | Apert | 6.9 | LF3 | Internal | No |
| 5 | Crouzon | 9.7 | MB | Internal | Yes |
| 6 | Crouzon | 4.2 | MB | Internal | No |
| 7 | Crouzon | 2.0 | MB | Internal | No |
| 8 | Crouzon | 6.1 | FB | Internal + external | No |
| 9 | Crouzon | 18.4 | MB | No distraction | No |

LF3 = Le Fort III, MB = monobloc, preop = preoperative

**Supplemental Table 4. Findings on preoperative and postoperative CT scans per preoperative and short-term facial photograph score**

|  |  | **Preop no clinical nasal deviation (n = 47)** | |  | **Preop clinical nasal deviation**  **(n = 9)** | |
| --- | --- | --- | --- | --- | --- | --- |
|  |  | **Short-term postop clinical nasal deviation** | |  | **Short-term postop clinical nasal deviation** | |
| **Findings CT scans** | **Total** | **Unchanged** | **More deviated** |  | **Unchanged** | **More deviated** |
| **No. patients** | 56 | 29 | 18 |  | 6 | 3 |
| **Preop anterior septum position** |  |  |  |  |  |  |
| No deviation | 23 (41) | 13 (45) | 8 (44) |  | 2/6 | 0/3 |
| Deviated | 33 (59) | 16 (55) | 10 (56) |  | 4/6 | 3/3 |
| **Median T3 (IQR), mos** | 4.0 (3.0 – 7.5) | 4.0 (3.0 – 9.0) | 4.5 (1.8 -7.8) |  | 2.5 (1.3 – 3.0) | 3.0 (1.5 – 3.5) |
| **Short-term postop increased anterior deviation** | 16 (29) | 6 (21) | 9 (50) |  | 0/6 | 1/3 |
| **Short-term postop increased posterior deviation** | 4 (7) | 2 (7) | 2 (11) |  | 0/6 | 0/3 |
| **No. patients with long-term postop CT** | 26 | 16 | 8 |  | 1 | 1 |
| **Median T4 (IQR), yrs** | 2 | 4.5 (2.9 – 9.0) | 5.3 (3.6 – 7.9) |  | 10.9 | 4.2 |
| **Long-term postop septal deviation** |  |  |  |  |  |  |
| Unchanged | 20 (77) | 13 (81) | 5/8 |  | 1/1 | 1/1 |
| Worsened | 1 (4) | 1 (6) | 0/8 |  | 0/1 | 0/1 |
| Improved | 5 (19) | 2 (13) | 3/8 |  | 0/1 | 0/1 |

Values represent number of patients (percentages).

Preop = preoperative, postop= postoperative, IQR = interquartile range, T3 = first postoperative CT scan, T4 = long-term postoperative CT scan
